# A Rational Approach to Drug Repositioning in β-thalassemia: Induction of Fetal Hemoglobin by Established Drugs

**DOI:** 10.12688/wellcomeopenres.17845.2

**Published:** 2022-06-23

**Authors:** Marco Prosdocimi, Cristina Zuccato, Lucia Carmela Cosenza, Monica Borgatti, Ilaria Lampronti, Alessia Finotti, Roberto Gambari

**Affiliations:** 1 Rare Partners srl Impresa Sociale, Via G.Boccaccio 20, Milano, 20123, Italy; 2 Center ‘Chiara Gemmo and Elio Zago’ for the Research on Thalassemia, Department of Life Sciences and Biotechnology, University of Ferrara, Via Fossato di Mortara 74, Ferrara, 44121, Italy

**Keywords:** Thalassemia; sirolimus; fetal hemoglobin; hydroxyurea; drug repositioning.

## Abstract

Drug repositioning and the relevance of orphan drug designation for β-thalassemia is reviewed. Drug repositioning and similar terms ('drug repurposing', 'drug reprofiling', 'drug redirecting', ‘drug rescue’, ‘drug re-tasking’ and/or 'drug rediscovery') have gained great attention, especially in the field or rare diseases (RDs), and represent relevant novel drug development strategies to be considered together with the “off-label” use of pharmaceutical products under clinical trial regimen. The most significant advantage of drug repositioning over traditional drug development is that the repositioned drug has already passed a significant number of short- and long-term toxicity tests, as well as it has already undergone pharmacokinetic and pharmacodynamic (PK/PD) studies. The established safety of repositioned drugs is known to significantly reduce the probability of project failure. Furthermore, development of repurposed drugs can shorten much of the time needed to bring a drug to market. Finally, patent filing of repurposed drugs is expected to catch the attention of pharmaceutical industries interested in the development of therapeutic protocols for RDs. Repurposed molecules that could be proposed as potential drugs for β-thalassemia, will be reported, with some of the most solid examples, including sirolimus (rapamycin) that recently has been tested in a pilot clinical trial.

## Abbreviations

HbF, fetal hemoglobin; HU, hydroxyurea; EMA, European Medicine Agency; BHT, bone marrow transplantation.

## Background

Despite the widespread view that research in rare disease is economically difficult, the lack of available treatments for many diseases and the facilitations offered by the present legislation, in particular legislation on orphan drugs that will be discussed in this paper later on, resulted in the fact that this field is now increasingly considered by small and large companies, including big pharma. Within this framework, repositioning is a fundamental process ^1– 3^ and this document will focus the attention on some recent advancements in the field of β-Thalassemia.

The β-thalassemias are a group of hereditary hematological diseases caused by more than 300 mutations of the human β-globin gene, causing low or absent production of adult β-globin and excess of α-globin content in erythroid cells, and causing ineffective erythropoiesis and low or absent production of adult hemoglobin (HbA) ^4^. Despite the fact that β-thalassemia is considered a rare disease in several countries, the syndrome is the most important patholog in developing countries ^5^. This has been associated with a lack of genetic counseling and prenatal diagnosis, largely contributing in the maintenance of a very high frequency within the population ^6^. In particular, β-thalassemia is prevalent in Mediterranean countries, the Middle East, Central Asia, India, Southern China, and the Far East as well as countries along the north coast of Africa and South America. The highest carrier frequency is reported in Cyprus (14%), Sardinia (10.3%), and Southeast Asia. Population migration and intermarriage between different ethnic groups has introduced thalassemia in almost every country of the world, including Northern Europe where thalassemia was previously absent. It has been estimated that about 1.5% of the global population (80 to 90 million people) are carriers of beta-thalassemia, with about 60,000 symptomatic individuals born annually, the great majority in the developing world. According to the Thalassemia International Federation, only about 200,000 patients with thalassemia major are alive and registered as receiving regular treatment around the world. In European Countries migration had changed and is changing the landscape, indeed a good estimate has been recently published with regard to the Italian situation ^7^.

The conventional treatment of patients affected by severe forms of β-thalassemia is based on regular blood transfusions and chelating therapy ^8, 9^. An appropriate regimen of transfusion therapy and optimal safety of transfused blood are key concepts in protocols developed for routine administration of red blood cells to patients with thalassemia ^10^. While the recent introduction of Luspatercept ^11, 12^ gives hopes to many patients, the only treatment that can be considered a cure for β-thalassemia is transplantation of hematopoietic stem-cells (BMT, bone marrow transplantation) ^13^. In this procedure, enhanced conditioning regimens on one hand and improved procedures for donor selection on the other led to significant better results when data obtained applying these methods were considered in recent years ^14^. In BMT, the donor is ideally a human leukocyte antigen (HLA)-identical sibling of the β-thalassemia patient to be transplanted. However, this is not frequent, as about 60–65% of the β-thalassemia patients lack such suitable BM donors. In this case, HLA-matched unrelated donors night be considered for BMT. In addition, the cost of BMT for the health systems is high and requires technical expertise and selected facilities ^15^.

More recently gene therapy has been studied with promising results. To this end various approaches can be used ^16^. Lentiviral vectors expressing the β-globin gene can be used to correct the phenotype of patients and genome and epigenome editing technologies are explored to alter globin gene regulation in order to reactivate HbF to mimic the protective effect of genetic traits typical of hereditary persistence of fetal hemoglobin (HPFH) condition. Finally, efficient CRISPR-Cas9-based genome editing of β-globin gene can also be obtained as demonstrated by Cosenza *et al*. ^17^

Alternatively, a growing evidence support the concept that induction of fetal hemoglobin (HbF) by pharmacological agents might be of great interest for the development of therapeutic protocols for β-thalassemia ^18– 20^. Induction of HbF has been the object of several studies and review papers ^21– 23^ and at this stage there are several compounds that reached the stage of clinical testing, including sirolimus (see later), benserazide and thalidomide ^20, 24^. In this context, orphan drug designation, in addition to patent applications, is considered a parallel strategy to help the pharmaceutical development of drugs to be used for therapeutic treatment of β-thalassemia. In this respect drug repositioning appears to be a strategy of major interest.

## Drug repositioning

**Figure 1.**
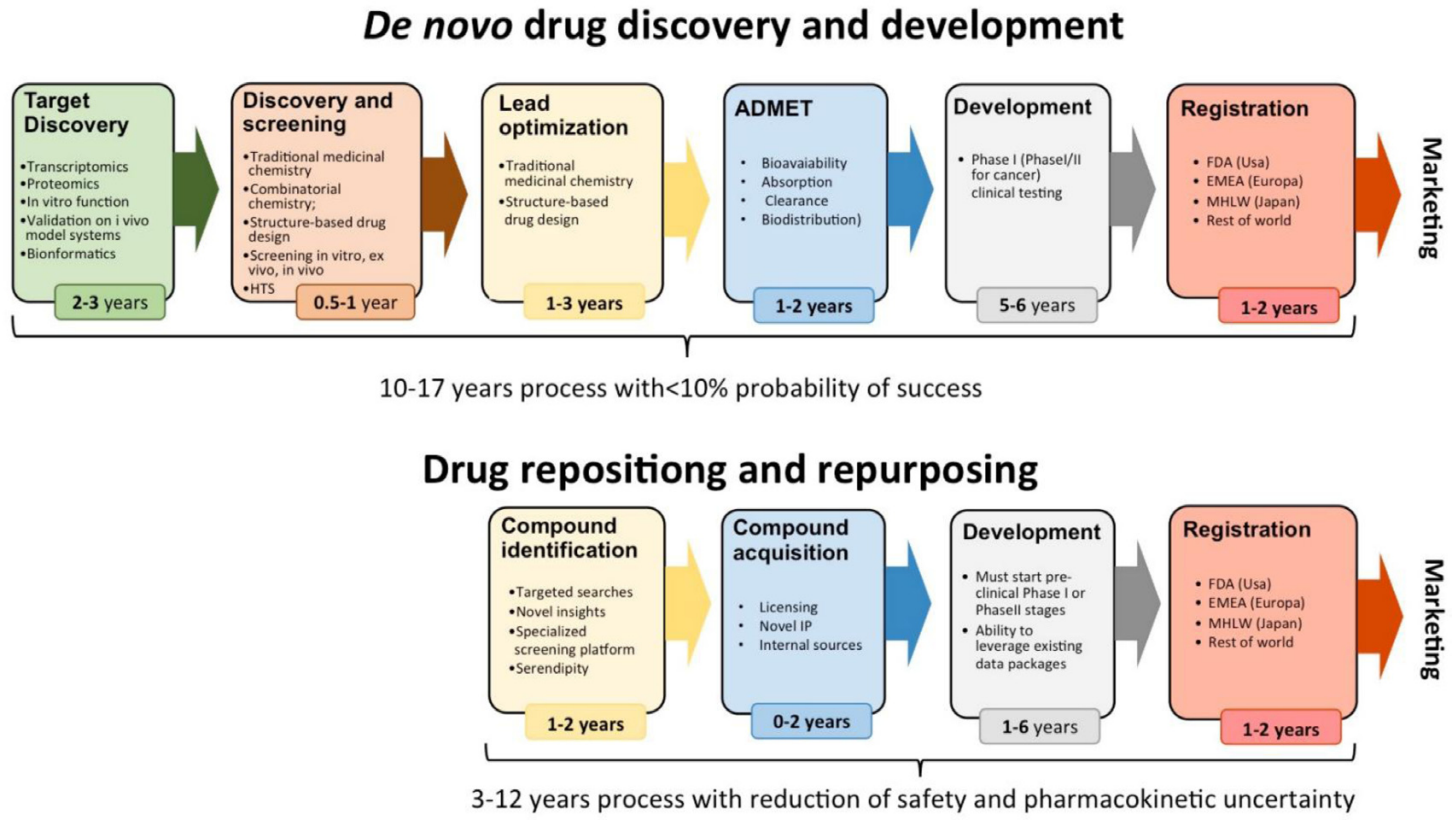
Comparison of time-to-market for new chemical entities (upper part of the Figure) versus repositioned drugs (lower part of the figure) (modified with permission from reference 1).

Drug repositioning is about finding novel indications for existing drugs ^1^. This approach has been always used empirically once a particular drug was in the market or in clinical trials, and there are many well-known examples of this approach, including those shown in Table 1. The main advantage of this approach is that it allows initiating regulatory studies on the repurposed indication. Actually, in certain cases it is possible to directly initiate Phase II clinical studies, thus shortening the development time up to 3 years instead of the 10–17 years standard within the industry. In 2004 Ashburn and Thor indeed published a very interesting paper on the subject and is now generally accepted that repositioning shortens the development time in at least 5 years. Figure 1 is taken from their paper.

**Table 1.** Selected examples of repositioning drugs in non-orphan indications.

Drug	Original indication (trade name; originator)	New indication (trade name; repositioner)	References
**Finasteride**	Benign prostatic hyperplasia (Proscar; Merck)	Hair loss (Propecia; Merck)	27
**Sildenafil**	Angina (N/A; Pfizer)	Male erectile dysfunction (Viagra; Pfizer)	28
**Thalidomide**	Sedation, nausea and insomnia (Contergan; Chemie Grunenthal)	Leprosy and multiple myeloma (Thalomid; Celgene)	29

In mid 2000’s the pharmaceutical and biotech industry recognized the repositioning as a valuable strategy to reduce costs and attrition rates, and efforts started to systematically evaluate known drugs into models of most prevalent diseases ^25^. In the case of rare diseases, this approach is hampered by the paucity of suitable disease models and, even more severely, by the paucity of financial resources enabling complete clinical development at affordable prices ^26^. 

Despite initial difficulties, later on several projects have been successfully completed and now many drugs, originally developed for different indications, are commonly applied in rare diseases. Some examples of successful repositioning projects are reported in Table 2, interestingly sildenafil, already mentioned in Table 1, is mentioned in this table as well.

**Table 2.** Selected examples of repositioning drugs in orphan indications.

Drug	Original indication (trade name; originator)	New indication (trade name; repositioner)	References
Sildenafil	Male erectile dysfunction (Viagra; Pfizer)	Pulmonary arterial hypertension (Revatio; Pfizer)	28
Miglustat	Mild-to-moderate type I Gaucher disease (Zavesca, Actelion)	Paediatric patients with Niemann-Pick type C disease (Zavesca, Actelion)	30
Sirolimus	Prophylaxis of organ rejection (Rapamune; Pfizer)	Lymphangioleiomyomatosis (Rapamune; Pfizer)	31

## Orphan drug designation

In the European Union Orphan Drugs are medicinal products intended for the diagnosis, prevention or treatment of rare diseases, which are diseases affecting less than one in 2,000 persons or a maximum of 250,000 citizens in the European Union. It is considered that pharmaceutical companies are unwilling to develop such drugs under normal market conditions, as the cost of bringing them to the market would not be recovered by the expected sales of drugs without providing incentives. The same concept has been applied in other countries, with minor differences, in particular on the prevalence of the disease, as shown in Table 3.

**Table 3.** Definition of rare disease in different regions.

Country, region or organisation	Relevant orphan drug legislation	Definition
**European Union**	Regulations (EC) No 141/2000 (the Orphan Regulation) and No 847/2000	Life-threatening or chronically debilitating conditions that affect no more than 5 in 10,000 people in EU
**WHO**	No specific legislation	A disease or a condition affecting 0.65–1 in 1000 inhabitants
**USA**	US Orphan Drug Act of January 1983 and amendments	A disease or a condition which affects fewer than 200,000 patients in US
**JAPAN**	Japan Law 145 - 10 August 1960 (revised in 1993)	A disease that affects fewer than 50,000 patients in Japan
**AUSTRALIA**	Therapeutic Goods Act in 1989 (Revised in 1997)	A disease that affects fewer than 2000 patients per year
**CANADA**	No specific legislation	Canada accepts WHO definition

In order to increase the probability that rare diseases are considered viable targets for drug development, a number of incentives are provided to companies willing to develop such drugs. In particular, Orphan Drugs Incentives include market exclusivity (6 – 10 years), tax credits, development grants, including possibility to apply for EU calls on rare diseases, and regulatory review (assistance and fast-track approval). Recently these incentives have been analyzed and it has been recognized that legal and regulatory hurdles are still limiting this opportunity and since patients often ask for quick responses, potential solutions to overcome these limitations have been proposed in order to increase the probability that drug companies are willing to be involved in the repositioning process for rare diseases ^32^.

**Figure 2.**
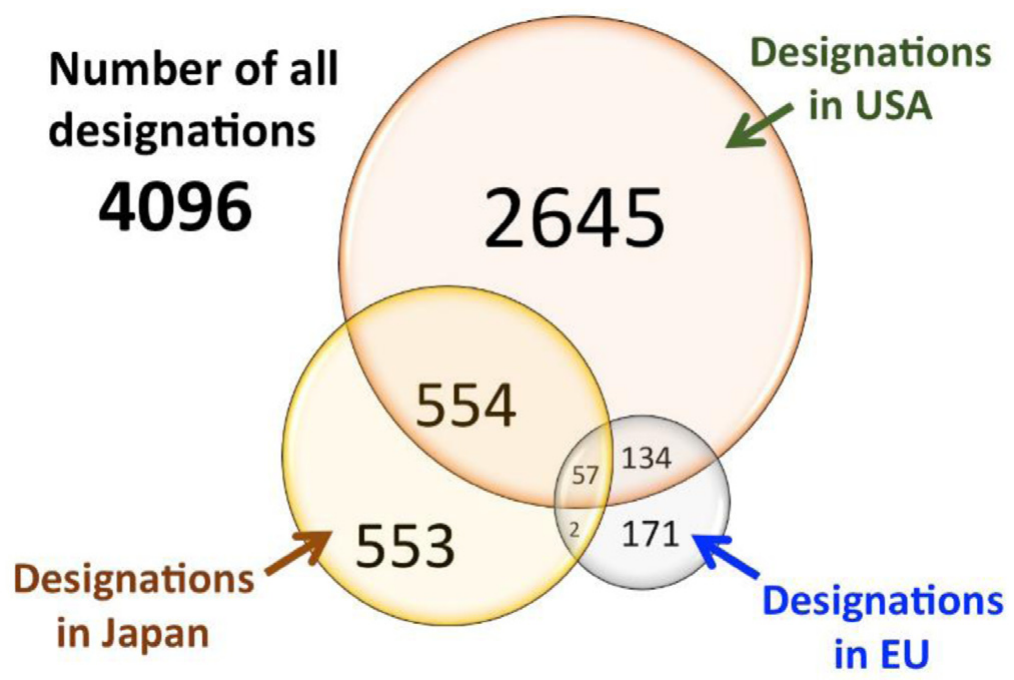
Venn diagram for orphan drug designations in the USA, EU, and Japan. Underlined data are the ‘matched data’, which comprise commonly designated products in USA/EU and USA/Japan (modified with permission from reference 33).

Probably as a consequence of the existence of incentives, the number of drugs designed as orphan has been constantly increasing in the last few years, substantially in all the countries. In a review ^33^ the trend has been extensively examined as far as Japan, EU and USA are concerned, reaching the conclusion that marked regional differences in the timing of designation, designation/approval ratios, applicant types, and drug types still exist. This is clearly visible in Figure 2, where is evident that many drugs were granted ODD in one Region but not in the other one and few of them were granted ODD in all the three regions.

**Figure 3.**
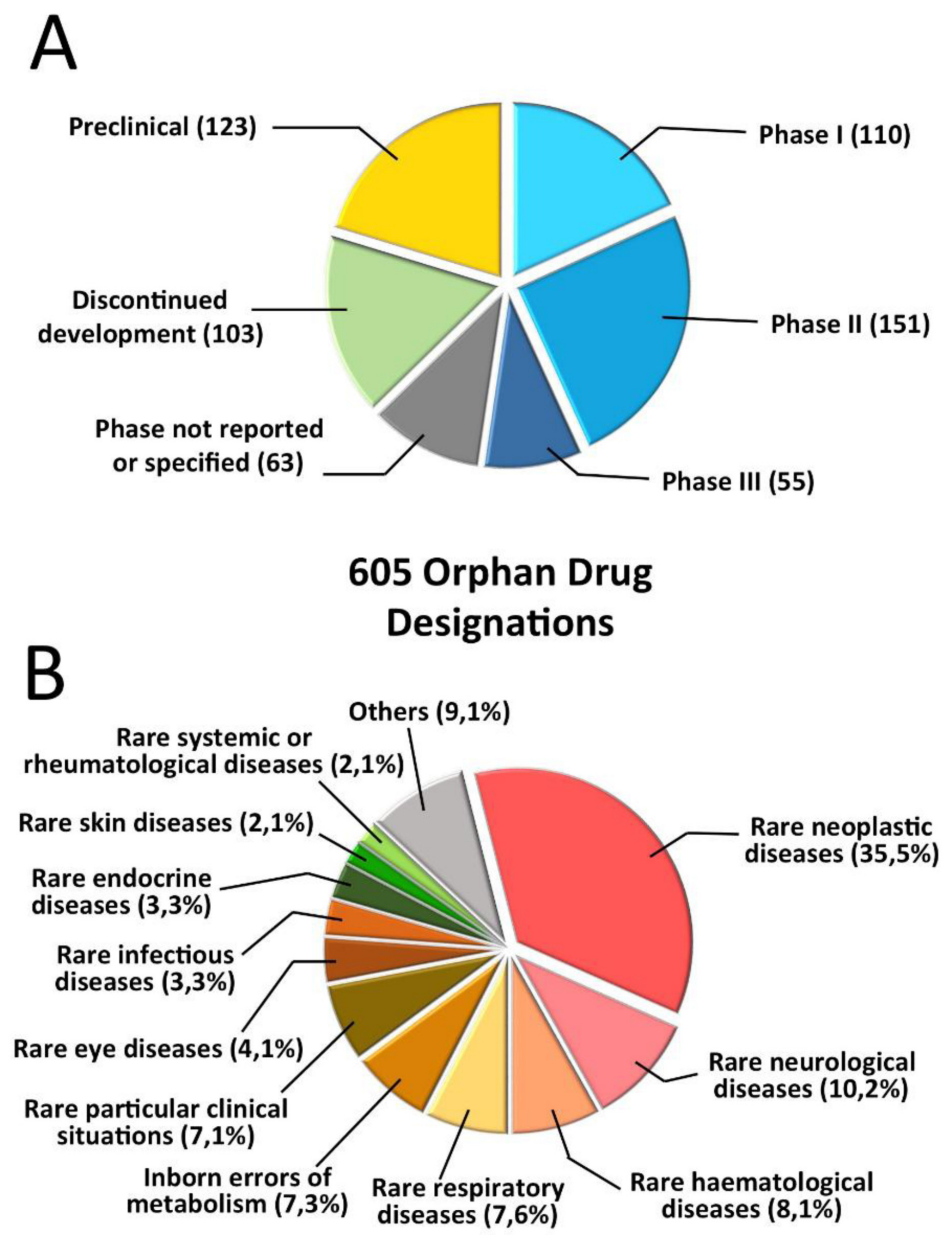
Analysis of orphan designations in the European Union. The data are for orphan designations granted between 2002 and 2012 with annual reports filed to the European Medicines Agency in 2013 or 2014 ( *n* = 605). **A** Orphan designations granted, subdivided by development stage **B** Orphan designations granted, subdivided by therapeutic area (modified with permission from reference 34).

Focusing the attention on EU, Morel et al performed an analysis of ODs granted between 2002 and 2012 for which an annual report was available in 2013 or 2014, with a sample of over 600 records ^34^. Figure 3, taken from the mentioned paper, describes the therapeutic areas (note that oncology is the most relevant area) and the development phase. Overall, according to the authors, the study findings confirm the success of the European regulatory framework in promoting therapeutic innovation for rare diseases, and in being a catalyst for SME growth.

It is important to mention that in EU several conditions are pre-requisite to obtain ODD. The drug indeed must be intended for the treatment, prevention or diagnosis of a disease that is life-threatening or chronically debilitating. Furthermore, the prevalence of the condition in the EU must not be more than five in 10,000 or it must be unlikely that marketing of the medicine would generate sufficient returns to justify the investment needed for its development. Finally, no satisfactory method of diagnosis, prevention or treatment of the concerned condition is authorized, or, if such a method exists, the medicine must be of significant benefit to those affected by the condition. This concept is more tricky than other ones, as it may be based on ‘clinically relevant advantage’ or a ‘major contribution to patient care’, obviously not yet demonstrated at an early stage of development. Interestingly, the concept of significant benefit has no equivalent outside the EU, thus approach of companies to Regulatory Authorities in inevitably different when dealing with procedures in different geographical areas. Fregonese *et al.* reviewed the evidence gathered in Europe in 15 years, reaching the conclusion that the review of the existing practices for the demonstration of significant benefit and the resulting conceptual framework could be useful for sponsors to reflect on potential areas of advantage of their candidate products ^35^. Some of the same authors recently published an updated analysis, considering that definition of a rare condition is becoming more and more challenging on the basis of technological advancements ^36^.

In general, a product can be granted Orphan Drug status by a Regulatory Authority after review of the application, presented by a sponsor, in many cases a Company. Applications to obtain ODD are somehow different in different regions, but in every case several conditions must be met. In particular, EU will grant ODD status, in some cases after a meeting with sponsor’s representatives, only if the application covers a number of subjects, including:

(a) description of the condition;

(b) proposed orphan indication;

(c) medical plausibility;

(d) justification of the life-threatening or debilitating nature of the condition;

(e) prevalence of the condition;

(f) other methods for diagnosis, prevention or treatment of the condition;

(g) potential for return on investment;

(h) description of the stage of development.

Medical plausibility is particularly important and constitutes, de facto, an external validation of the rational of the project.

## Drug repositioning for rare diseases: β-thalassemia

It is well known that β-thalassemia has a peculiar geographic distribution ^5^ and in some areas its prevalence is relatively high, nonetheless the disease qualifies as a rare disease in USA, EU and Japan, thus designation can be obtained in all these regions. It this paper the attention will be focused only on the possibility to treat β-thalassemia though an action on fetal hemoglobin considering also the most appropriate end point to be analyzed in clinical studies.

It is important to mention that HU, despite the lack of specific approval for β-thalassemia and of robust clinical evidence ^37^, is indeed used and provides sustained benefits in certain patients ^38^. On the other hand, a substantial percentage of patients does not respond and some of the responding ones become insensitive after repeated administrations ^39^, thus new interventions are eagerly needed.

In general terms, a repositioning effort to increase HbF in β-thalassemia seems highly promising, on the basis of the following considerations:

(a) there is a clear clinical pharmacology target, increase of HbF, measurable with high reproducibility by several methods in patients ^40^;

(b) there is robust evidence that, at least for HU *, in vitro* response predicts *in vivo* response ^41^;

(c) there is little or no doubt that a positive result in terms of clinical pharmacology (increase of HbF) will reduce transfusion burden, the final goal of every project in the field ^18, 42^. 

Given the fact the HbF increase can be obtained in vitro by many agents already used for other indications, the main point is how to select the most promising agents and how to validate the rational supporting clinical trials. Patients not responding (or no more responding) to HU have the highest medical need, thus they probably are a group who deserve maximal priority. 

With regard to compounds that can be tested, there are a number of potential treatments ^21, 43^, but in order to proceed immediately toward clinical development, priority must be given to agents that can be rapidly given to thalassemia patients, on the basis of safety considerations firstly. In our view, the most relevant compounds in a “repositioning campaign” are those that can be given rapidly to thalassemia patients thanks to prior registration in other indications. To this end, as far as compounds under active development are concerned, thalidomide, benserazide and mipativat are particularly relevant.

Thalidomide is a drug approved for multiple myeloma in USA and EU and have been granted by FDA and by EMA the status of Orphan Drug for several indications, not including thalassemia. Thalidomide has previously been mentioned as a drug repositioned with success and may be useful in thalassemia patients as well. A recent meta-analysis ^44^, reporting the data from 12 trials with a total of 451 thalassemia patients, mostly of Chinese origin, concluded that thalidomide is a relatively safe and effective therapy to reduce the blood transfusion requirements and to increase Hb level in patients with β-thalassemia. This interesting finding follows many papers on the subject, including an early publication ^45^, suggesting that thalidomide may indeed have a place in thalassemia therapy. Furthermore, a pediatric study has been recently reported ^46^ showing a positive effect in children as well. Thalidomide is also studied in combination with HU ^47^, pointing out the relevance of combination treatment in rare diseases. Needless to say, safety must be the primary concern for such a drug.

Benserazide hydrochloride was designated as Orphan Drug for thalassemia by EMA in December 2014. To the best of our knowledge FDA never granted ODD status to this drug. Benserazide has been used chronically in humans to inhibit amino acid decarboxylase to enhance plasma levels of L-dopa. This compound demonstrated > 20-fold induction of γ-globin mRNA expression in anemic baboons and increased F-cell proportions by 3.5-fold in transgenic mice ^48^. Pace *et al*. ^49^ recently reported that intermittent treatment with benserazide in a mice model significantly increased proportions of red blood cells expressing HbF and HbF protein per cell. Additionally, orally administered escalating doses of benserazide in an anemic baboon induced γ-globin mRNA up to 13-fold and establish an intermittent dose regimen for clinical studies. On the basis of these data a clinical trial named "The BENeFiTS Trial in Beta Thalassemia Intermedia, NCT04432623" has been launched and is ongoing. This trial will first evaluate three dose levels in small cohorts of non-transfused patients with β-thalassemia intermedia. The most active dose will then be evaluated in larger subject groups with β-thalassemia and other hemoglobinopathies, such as sickle cell disease.

Along this line, two compounds were selected by our group for further consideration, resveratrol ^50^ and sirolimus. Resveratrol is considered a food supplement; thus it is not rational to develop it as a drug. On the other hand, sirolimus is commercialized since many years ago in a different indication (prevention of kidney transplant rejection) in USA and in EU, thus it is possible to apply to Regulatory Authorities in order to get ODD. Sirolimus in vitro is a strong inducer of HbF synthesis, as shown by in different labs ^51– 53^. In an effort for bringing sirolimus to possible clinical evaluation, applications were filed in EU and in USA, obtaining ODD designation by EMA (Treatment of β-thalassaemia intermedia and major, December 2015) and by FDA (Treatment of β-thalassemia, June 2016). Apart from the data generated by in vitro studies, several groups provided evidence supporting the use of this established drugs in β-thalassemia through experiments in animal model ^54– 57^ and reporting an increase level of HbF in clinical settings ^58, 59^.

Interestingly, our group is further exploring the possibility of repositioning old drugs as new drugs for thalassemia, as shown by the recent study who identified Cinchona alkaloids (cinchonidine, quinidine and cinchonine) as natural HbF-inducing agents in human erythroid cells ^60^. Two highly active compounds, cinchonidine and quinidine, were able to induce γ-globin mRNA and HbF in erythroid progenitor cells isolated from β-thalassemia patients. The data obtained demonstrate that cinchonidine and quinidine are potent inducers and HbF in erythroid progenitor cells isolated from nine β-thalassemia patients. The data obtained strongly indicate that these compounds deserve consideration in the development of pre-clinical approaches for therapeutic protocols of β-thalassemia. These compounds should be considered as repurposed drugs, as quinidine has been employed in a variety of cardiac complications, such as arrythmias, atrial fibrillation, idiopathic ventricular fibrillation, Brugada syndrome, and Short QT syndrome ^61– 63^.

Finally, even if it is not primarily a compound acting on HbF, Mitapivat deserves attention since this drug, after Priority Review, has been approved as first disease-modifying therapy for hemolytic anemia in adults with pyruvate kinase deficiency by FDA in February 2022. FDA granted ODD status for treatment of thalassemia to the compound on August 2020. Mipativat (formerly known as AG-348), an allosteric activator of RBC-specific pyruvate kinase, represents a distinct and novel mechanism. RBC-specific pyruvate kinase activation increases adenosine triphosphate synthesis, as well as reduces the production of reactive oxygen species and concentration of 2,3-diphosphoglycerate. After successful phase two trials, phase three studies of mitapivat in TDT (NCT04770779) and NTDT (NCT04770753) patients are under way.

## Sirolimus as a drug candidate for β-thalassemia

As previously mentioned, Sirolimus has been granted ODD status for thalassemia by both the EMA (EU/3/15/1585) | European Medicines Agency (europa.eu) and FDA (Search Orphan Drug Designations and Approvals (fda.gov)

With the idea that sirolimus could be a drug to be tested soon in patients, experimental evidence supporting key points relevant to clinical development have been obtained. Some of these points will be discussed here.

As previously discussed, one key point is the availability of a reproducible *in vitro* test potentially able to predict in vivo response. To this end, recent findings reported by Cosenza *et al*. demonstrated that HbF induction can be studied reproducibly in thalassemia patients also after transporting biological samples from a lab to another one, thus making multicenter trial a possible option ^40^.

**Figure 4.**
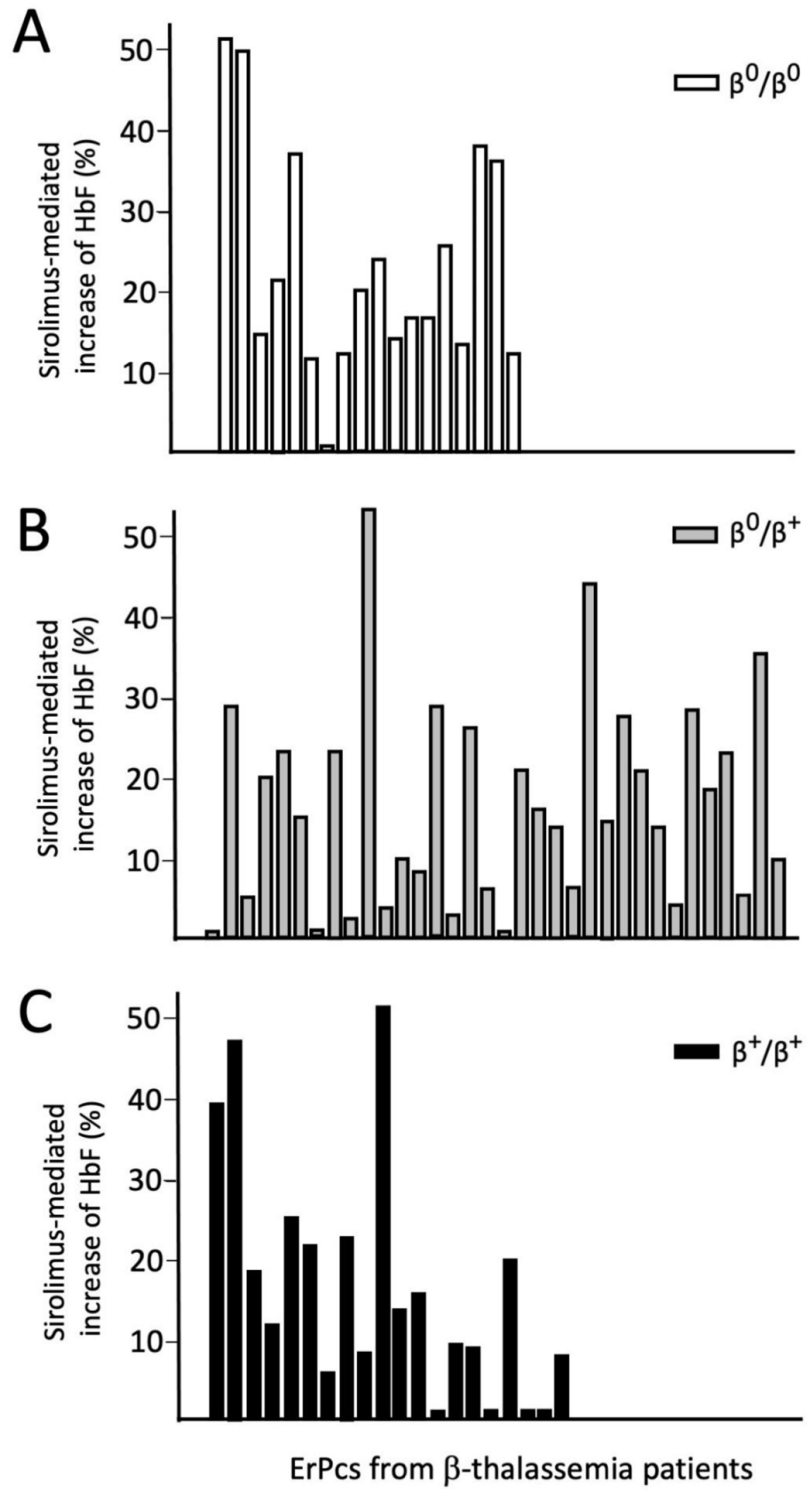
Analysis of sirolimus-mediated increase of HbF (% of total hemoglobin analyzed by HPLC) in patients with β ^0^/β ^0^, β ^+^/β ^0^ and β ^+^/β ^+^ genotypes (Zuccato, Cosenza and Gambari, personal communication).

Another key point is the percentage of patients where a consistent response may be expected on the basis of preliminary in vitro test and, along the same line, the possibility that patients not responding to HU may respond to a different agent. In order to define a patient as a responder we applied the concept that an increase of more than 20% in the HbF level induced in vitro by 100 nanomolar sirolimus is considered a positive response. Figure 4 shows data relative to 72 ErPC cultures from patients with a β ^0^/β ^0^, β ^+^/β ^0^ and β ^+^/β ^+^ genotypes (18, 34 and 20 ErPC cultures, respectively). A positive response was observed in 41.7 % samples.

A comparison with response to hydroxyurea (HU) was done for more than 37 β-thalassemia patients, observing that sirolimus was able to induce HbF in 46.15% of the cultures not responsive to HU. In addition, sirolimus displayed higher efficiency than HU in 57.14% of the cultures responsive to both sirolimus and HU. These observations pave the way to a step we believe may change to landscape of clinical trials in this field, namely the selection of patients to be treated by a drug on the basis of a pre-trial test performed *in vitro*. It is reasonable to hypothesize that a patient whose Erythroid Precursor Cells (ErPCs) are highly responsive *in vitro* will be more responsive in vivo, with a potential benefit exceeding side effect expectations. In other words, a first analysis of the in vitro response is suggested to identify patients to be selected for a possible clinical trial as the ones with the highest probability to be responder

## Ongoing activities toward clinical application of experimental findings

On the basis of these concepts, clinical development for sirolimus in thalassemia has been planned and discussed with EMA with regard to the first phases, in particular pilot trials. Two main concepts should be outlined as fundamental for clinical development in this pathological condition, namely HbF as a surrogate exploratory end point and the possibility of selection of “potentially responsive patients” on the basis of data obtained exposing cells from each patient to sirolimus in vitro before giving the drug by the oral route.

After obtaining EMA advice we actively pursued the possibility of running exploratory clinical trials in collaboration with clinicians involved in patient’s management ^64^. As a result, two clinical trials have been conducted with sirolimus on β-thalassemia patients. The first one, named Sirthalaclin, has been completed (NCT03877809, A Personalized Medicine Approach for β-thalassemia Transfusion Dependent Patients: Testing sirolimus in a First Pilot Clinical Trial) and the second one, named Thala-Rap is ongoing (NCT04247750, Treatment of β-thalassemia Patients with Rapamycin (Sirolimus): From Pre-clinical Research to a Clinical Trial). Both trials are based on the use of low-dosages of the repurposed drug sirolimus (rapamycin) for a 12 months period.

The main objective of these interventional, pilot, open-label phase II studies with sirolimus II in patients with β-TDT (transfusion-dependent thalassemia) was to verify its efficacy as in vivo HbF inducer aiming to the reduction of the transfusions need with an overall good tolerability. In addition, the possibility to know genotypes and DNA polymorphisms (including HbF-associated polymorphisms) is expected to bring important information for a possible personalized and precision medicine approach in β-Transfusion Dependant Thalassemia (TDT).

The concept has been spelled out in the clinical trials, where cells from patients has been initially exposed in vitro to sirolimus and then only patients with high probability of response has been treated in vivo. In our view, application of such a procedure may be considered as a step toward personalized medicine in rare diseases, most likely a fundamental step for drug development where only few patients can be studied and thus strict selection criteria greatly increase the probability of a positive outcome.

As mentioned previously, the results of Sirthalaclin have been recently analyzed and the main finding has been the observation that was that expression of γ-globin mRNA was increased in blood and erythroid precursor cells isolated from β-thalassemia patients treated with low-dose sirolimus. A second important conclusion of our trial was that sirolimus influences erythropoiesis and reduces biochemical markers associated to ineffective erythropoiesis (I.E.) (excess of free α-globin chains, bilirubin, soluble transferrin receptor and ferritin). In most (7/8) of the patients a decrease of the transfusion demand index was indeed observed. The drug was well tolerated with minor effects on immunophenotype, the only side effect being frequently occurring stomatitis ^65^.

Even if sirolimus has been tested primarily on the basis of the observed increase of HbF, the clinical experience, admittedly limited, indicated that other parameters may be appropriate as end points for clinical trials. In general, it has been considered that γ-globin mRNA transcripts and HbF production are moving in parallel after therapeutic interventions.

This observation has been confirmed in Sirthalaclin trial, however mRNA measurement seems to be more precise than blood HbF measurement, thus we suggest that this parameter should be considered fundamental for future trials, also bearing in mind that samples can be easily shipped from a clinical center to a centralized laboratory located in a different country. Another point for future trials with sirolimus is related to the frequent occurrence of transfusions in TDT patients. Having a drug potentially active as a corrector of deficient erythropoiesis the interference of transfusions may be avoided, or limited, studying sirolimus in NTDT patients where blood transfusions are only employed rarely.

## Discussion

Induction of HbF by drugs in patients with β-thalassemia is coming close to clinical proof of concept trials, with sirolimus and with other repositioned products as well. The trials should be able to contribute toward responses to fundamental questions, in particular we should know, in a relatively short time, if the results observed *in vitro* and in experimental animals translate in meaningful HbF increase in humans with β-thalassemia. Further, the possible increase in HbF may or may not translate in a better clinical outcome; contributing to the response to this question will be a major advancement in therapy design.

## Declarations

Author contribution: Conceptualization, M.P., C.Z., L.C.C. and R.G; methodology, I.L., M.B. and A.F.; investigation, C.Z., L.C.C. and I.L.; writing—original draft preparation, M.P., C.Z., L.C.C. and R.G; writing–review and editing, M.P. and R.G.
