# Supplementary material for: A Rational Approach to Drug Repositioning in β-thalassemia: Induction of Fetal Hemoglobin by Established Drugs
Source: Wellcome Open Res. 2022 Aug 17;7:150. Originally published 2022 May 12. [Version 3] doi: 10.12688/wellcomeopenres.17845.3 (PMC9453112; doi:10.12688/wellcomeopenres.17845.3)
Supplement: Peer review history

# Peer review: Reviewer response for version 3

**DOI:** 10.21956/wellcomeopenres.20147.r52056

**Published:** 2022-09-07

**Authors:** Alan Talevi

**Affiliations:** 1 Laboratory of Bioactive Compounds Research and Development (LIDeB), Department of Biological Sciences, Faculty of Exact Sciences, National University of La Plata (UNLP), La Plata, Argentina

The authors have satisfactorily covered most of my previous comments. Thank you for that. I have just only one comment to make about the revised version.

 In one of my original comments, I indicated that "Authors discussed that already approved drugs should be given priority, as they are more likely to be more directly repurposed. Is that the only prioritisation criterion that they consider? The scenario is the same for approved drugs that are delivered systemically that those used topically, for instance? What about compounds that are originally used in high doses compared with those given in very low doses? Also, don't the authors think that pharmaceutical companies would find economic incentives in rescuing a drug candidate that was abandoned due to a lack of sufficient efficacy for the original pursued indication?".

 Authors responded to this comment by inserting a series of sentences in the "Orphan drug designation" section, namely "As discussed in another section, drugs already studied are particularly suited for rare diseases application. A fundamental point in any development project is the interest of a drug company in the product. This applies to drugs to be given by any administration route and for drugs for rare diseases also". 

 What I meant in my original comment was that "approved" status is not a sufficient condition for a drug to be straightforwardly repurposed. For instance, a drug may have been previously approved for topical treatments and intended to be repurposed for systemic treatment, but in that case, much of the regulatory preclinical and clinical development should be performed anyway, despite the drug is already or was already approved for other indication. On the other hand, dose compatibility between the new indication and the previous one may help bypass some clinical studies, although the new indication requiring different dose strengths might be commercially attractive to enforce the new indication, if approved. Finally, a pharmaceutical company will possibly prioritize repurposing / rescuing drugs in its own portfolio rather, even if they did not achieve approved status yet, rather than repurposing drugs from other companies (due to freedom to operate, commercial reasons, etc.). 

 In other words, my original comment focused on the fact that which repurposing candidates should be prioritized highly depends on the context, and not only on prior approval. In some cases, as discussed, repurposing a failed or not-approved-yet drug could be convenient.

Is the review written in accessible language?

Yes

Are all factual statements correct and adequately supported by citations?

No

Are the conclusions drawn appropriate in the context of the current research literature?

Yes

Is the topic of the review discussed comprehensively in the context of the current literature?

Yes

Reviewer Expertise:

drug discovery and drug design, computer-aided drug repurposing, neglected and rare diseases

I confirm that I have read this submission and believe that I have an appropriate level of expertise to confirm that it is of an acceptable scientific standard.

# Peer review: Reviewer response for version 2

**DOI:** 10.21956/wellcomeopenres.19935.r51414

**Published:** 2022-07-28

**Authors:** Alan Talevi

**Affiliations:** 1 Laboratory of Bioactive Compounds Research and Development (LIDeB), Department of Biological Sciences, Faculty of Exact Sciences, National University of La Plata (UNLP), La Plata, Argentina

The article discusses the potential use of repurposed drugs for thalassemia, providing some general background on drug repurposing and orphan drugs. The article is of interest, although some points should be, in my opinion, addressed before indexing. 

- I suggest changing "repositioning" to "drug repositioning" in the background section. 
- Please revise the following sentence: "the syndrome is the most important patholog in developing countries". On the one hand, I think the authors intended to say "pathology". On the other, I believe the notion that thalassemia is THE MOST important pathology in developing countries is extremely debatable.
-  I believe "always" should be replaced by "initially" or "in the past", in the following sentence "This approach has been always used empirically once a particular drug was in the market or in clinical trials". Moreover, the information shown in Table 1 is too brief to justify a Table. Either include these examples in the main text only or enrich the Table somehow (add more examples, more columns...). ,
- Please revise the grammar in "In European Countries migration had changed and is changing the landscape". I think "countries" should be without capital letters and "had changed" should be replaced by "has changed". 
- Please provide references supporting the following statement "However, this is not frequent, as about 60–65% of the β-thalassemia patients lack such suitable BM donors. In this case".  
- Table 1 is not well justified, as the information it contains is too brief, and only very few examples are presented. Please enrich the Table somehow or include this information directly into the main text. The same applies for Table 2. 
- The following statement could be misleading "This approach has been always used empirically once a particular drug was in the market or in clinical trials,", as preclinical validation of efficacy for the new indication is mandatory for repurposed candidates.
- It is said that "n 2004 Ashburn and Thor indeed published a very interesting paper on the subject and is now generally accepted that repositioning shortens the development time in at least 5 years" but none of this is supported by references.
- Please consider splitting the following sentence: "in some areas its prevalence is relatively high, nonetheless the disease qualifies as a rare disease in USA, EU, and Japan, thus designation can be obtained in all these regions.".
- Authors discussed that already approved drugs should be given priority, as they are more likely to be more directly repurposed. Is that the only prioritisation criterion that they consider? The scenario is the same for approved drugs that are delivered systemically that those used topically, for instance? What about compounds that are originally used in high doses compared with those given in very low doses? Also, don't the authors think that pharmaceutical companies would find economic incentives in rescuing a drug candidate that was abandoned due to a lack of sufficient efficacy for the original pursued indication?
- Please revise the grammar carefully. Some examples "the recent study who identified"; "since many years".
- Maybe the authors should include a declaration of conflicting interests, as they are actively involved in the clinical development of one of the repurposed candidates on which the article focuses more clearly. 

Is the review written in accessible language?

Yes

Are all factual statements correct and adequately supported by citations?

No

Are the conclusions drawn appropriate in the context of the current research literature?

Yes

Is the topic of the review discussed comprehensively in the context of the current literature?

Yes

Reviewer Expertise:

drug discovery and drug design, computer-aided drug repurposing, neglected and rare diseases

I confirm that I have read this submission and believe that I have an appropriate level of expertise to confirm that it is of an acceptable scientific standard, however I have significant reservations, as outlined above.

# Response

**Published:** 2022-08-03

**Authors:** Marco Prosdocimi

In order to respond to the points made by the reviewer, appreciated by the authors, table 1 and table 2 have been merged and now they are just one table.

 The reviewer pointed out that quoted references did not support our view on the percentage of β-thalassemia patients lacking suitable BM donors. In order to respond, we modified the sentence and the quoted reference.

 Discussing criteria for industry viability for the repurposed drug we inserted a series of sentences at the end of the section on Orphan Drug Designation. In this section, we also mentioned that not only drugs given by the oral route can be repositioned.

 The potential conflict of interest by the first author is now clearly indicated.

 Many typesetting and minor points have been corrected according to the reviewer's comments

# Peer review: Reviewer response for version 1

**DOI:** 10.21956/wellcomeopenres.19758.r50666

**Published:** 2022-06-06

**Authors:** Yogen Saunthararajah

**Affiliations:** 1 Department of Translational Hematology and Oncology Research, Cleveland Clinic, Cleveland, OH, USA

Well-written review on an important approach to addressing clinical unmet needs in rare or orphan diseases such as beta-thalassemia.

 I have the following comments:

- As the authors acknowledge even in the abstract, for industry viability the repositioned drug will require intellectual property protection – it would be useful to the readership if the authors could summarize some of the alternative strategies that have been used to achieve this, and how they believe they may or may not work for repositioning efforts in beta-thalassemia
- It would be useful for readers if the authors used a table to summarize the candidate drugs they believe can be repositioned for beta-thalassemia, perhaps with pros and cons for each

Is the review written in accessible language?

Yes

Are all factual statements correct and adequately supported by citations?

Yes

Are the conclusions drawn appropriate in the context of the current research literature?

Yes

Is the topic of the review discussed comprehensively in the context of the current literature?

Yes

Reviewer Expertise:

epigenetics, sickle cell, thalassemia, myeloid malignancies

I confirm that I have read this submission and believe that I have an appropriate level of expertise to confirm that it is of an acceptable scientific standard.

# Response

**Published:** 2022-06-15

**Authors:** Marco Prosdocimi

We appreciate the comments of the reviewer and fully agree with his suggestions.

 We plan to submit a revised version of the manuscript incorporating suggestions from all the reviewers. For sure we will summarize possible strategies for intellectual protection and we will prepare a table with candidate drugs. An update will also be made on FDA and EMA web sites regarding possible new designations.

# Response

**Published:** 2022-08-03

**Authors:** Marco Prosdocimi

In order to respond to the points made by the reviewer, appreciated by the authors, a new table has been added, now this is table 3. Introducing the table, we also inserted a sentence explaining why each repurposed drug have pros and cons.

 Discussing criteria for industry viability for the repurposed drug we inserted a series of sentences at the end of the section on Orphan Drug Designation.
